# Recent advances of m^6^A methylation modification in esophageal squamous cell carcinoma

**DOI:** 10.1186/s12935-021-02132-2

**Published:** 2021-08-10

**Authors:** Xiaoqing Zhang, Ning Lu, Li Wang, Yixuan Wang, Minna Li, Ying Zhou, Manli Cui, Mingxin Zhang, Lingmin Zhang

**Affiliations:** 1 Department of Gastroenterology, The First Affiliated Hospital of Xi’an Medical University, No. 48 Feng Hao West Road, Xi’an, 710077 Shaanxi China; 2 Department of Scientific Research, The Affiliated Hospital of Xi’an Medical University, Xi’an, Shaanxi China; 3 Shaanxi University of Traditional Chinese Medicine, Xianyang, 712046 Shaanxi China; 4 Department of Anesthesiology, First Affiliated Hospital, Xi’an Jiaotong University, No. 277 Yanta West Road, Xi’an, 710061 China

**Keywords:** N6-methyladenosine, Methylation, Esophageal squamous cell carcinoma

## Abstract

In recent years, with the development of RNA sequencing technology and bioinformatics methods, the epigenetic modification of RNA based on N^6^-methyladenosine (m^6^A) has gradually become a research hotspot in the field of bioscience. m^6^A is the most abundant internal modification in eukaryotic messenger RNAs (mRNAs). m^6^A methylation modification can dynamically and reversibly regulate RNA transport, localization, translation and degradation through the interaction of methyltransferase, demethylase and reading protein. m^6^A methylation can regulate the expression of proto-oncogenes and tumor suppressor genes at the epigenetic modification level to affect tumor occurrence and metastasis. The morbidity and mortality of esophageal cancer (EC) are still high worldwide. Esophageal squamous cell carcinoma (ESCC) is the most common tissue subtype of EC. This article reviews the related concepts, biological functions and recent advances of m^6^A methylation in ESCC, and looks forward to the prospect of m^6^A methylation as a new diagnostic biomarker and potential therapeutic target for ESCC.

## Introduction

Esophageal cancer (EC) is one of the most invasive malignant tumors of digestive tract in the world, and its morbidity and mortality are still high in China [1]. 90% of the histopathological types of esophageal cancer are esophageal squamous cell carcinoma (ESCC) [2]. EC is caused by a variety of causes, among which genetic and epigenetic modifications play a key role in the occurrence and development of ESCC [3, 4]. In recent years, although surgical resection, combined radiotherapy and chemotherapy have improved the prognosis of patients with ESCC, the 5-year overall survival rate is still very low [5], between 20 and 30% [6]. Therefore, there is an urgent need to find new diagnostic biomarkers and potential therapeutic targets for ESCC patients.

In recent years, with the continuous development of tumor epigenetics, N6-methyladenine (m^6^A) has not only initiated a new era of post-transcriptional gene regulation in eukaryotes, but also rapidly become a research hotspot in the field of RNA methylation modification. As a reversible RNA methylation modification, m^6^A methylation is dynamically regulated by a variety of regulatory factors [7]. The imbalance of m^6^A methylation regulators changes the biological functions of cell proliferation, migration and invasion, and finally leads to the occurrence and development of tumor.

This article reviews the related concepts and biological functions of m^6^A methylation and the research progress in ESCC. In addition, it also emphasizes the prospect of m^6^A methylation as a new diagnostic biomarker and potential therapeutic target for ESCC.

## Related concepts and biological functions of m^6^A methylation

**Fig. 1:**
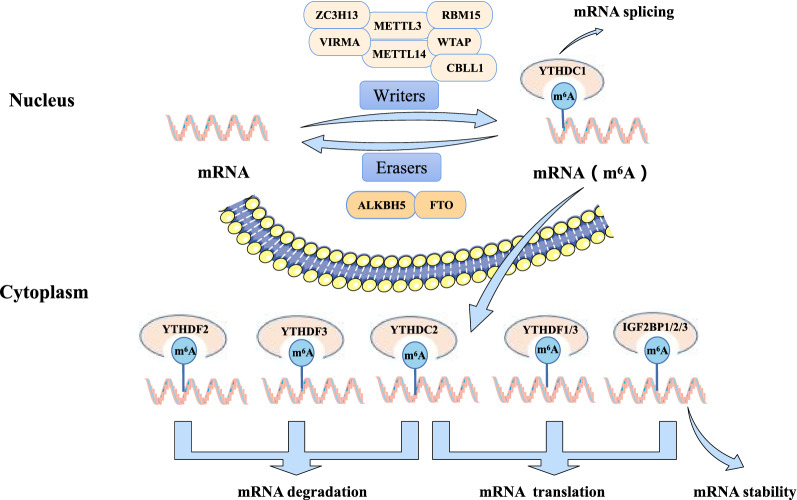
Basic mechanism of m^6^A methylation in RNA. Basic mechanism of m^6^A. The m^6^A methylation is catalyzed by the writer complex including METTL3, METTL14, WTAP, VIRMA, RBM15, ZC3H13 and CBLL1. The m^6^A modification is removed by demethylase FTO or ALKBH5. Reader proteins recognize m^6^A and determine target RNA nature

m^6^A refers to the methylation at the N6 position of adenosine, which is mainly concentrated in the 3'untranslated region near the mRNA Terminator. m^6^A modification mainly occurs in the RRm^6^ACH sequence [8]. As one of the most common and abundant internal modifications in mammals and eukaryotes [9], m^6^A RNA modification involves almost all aspects of RNA metabolism [10]. Its regulation process is mainly dynamically and reversibly regulated by a variety of regulatory factors. The methylated proteins involved in m^6^A methylation are mainly methyltransferases, demethylases and RNA binding proteins [11–13]. They can add, remove or give priority to recognize m^6^A sites, which play a key regulatory role in the expression of the whole genome and have a great impact on normal physiological function or pathological status [14].

m^6^A is involved in many cellular RNA processes, including transcription, splicing, nuclear transport, translation and degradation [12, 15]. By affecting the stability and half-life of mRNA, m^6^A regulates gene expression and regulates important biological functions such as mammalian reproductive function, circadian rhythm, adipogenesis and human lifespan [16]. Its interference may affect gene table regulation and cell biological function [17], and indirectly affect the stability and half-life of mRNA, leading to tumors and many diseases. The basic mechanism of m^6^A methylation in RNA is shown in Fig. 1.

### m^6^A modified writing gene (writers)

The written genes are various methyltransferases that promote m^6^A RNA methylation modification. At present, the components identified by m^6^A methyltransferase include methyltransferase like 3 (METTL3), METTL14, Wilms' tumor 1 associated protein (WTAP), and Virilizer like m^6^A methyltransferase associated protein (VIRMA/KIAA1429), RNA binding motif protein 15 (RBM15), zinc finger CCCH domain-containing protein 13 (ZC3H13), Casitas B-lineage lymphoma-transforming sequence-like protein 1, CBLL1/HAKAI).

Methyltransferase mainly forms stable complexes through core proteins such as METTL3, METTL14 and WTAP. m^6^A methylation occurs on the bases of mRNA [18], in which METTL3 is a subunit with catalytic activity, METTL14 is responsible for recognizing the substrate, WATP is mainly responsible for assisting METTL3 and METTL14 to target to nuclear spots, and WTAP has independent methylation sites, which can make some m^6^A sites specific methylation. VIRMA/KIAA1429, another component of methyltransferase complex, is a protein involved in alternative splicing and interacts with WTAP.

### m^6^A modified eraser gene (erasers)

The erase gene can remove the m^6^A modification in the RNA molecule by encoding m^6^A demethylase, which is the key to the reversible process of m^6^A modification [19]. m^6^A demethylase has been identified as AlkB homolog 5 (ALKBH5) [20] and fat mass and obesity associated (FTO) [21]. Although they have similar functions, but the action process is different.

ALKBH5 can catalyze the m^6^A modification demethylation of RNA in vitro [20], which can significantly affect the output of mRNA and RNA metabolism as well as the assembly of mRNA processing factors in nuclear spots. FTO has the function of demethylation of m^6^A on single-stranded DNA and RNA, and they are highly expressed in fat, brain and hypothalamus [22], which is vital to metabolism.

### m^6^A modified reading protein (readers)

The protein that selectively binds to the post-transcriptional product of m^6^A is called m^6^A reading protein. At present, the research on m^6^A modified reading protein is mainly focused on the YT521-B homology domain family (YTHDF), YT521-B homology domain containing (YTHDC), heterogeneous nuclear ribonucleoprotein binding protein (HNRNP), and insulin like growth factor 2 mRNA binding protein 2 (IGF2BP2), eukaryotic translation initiation factor (eIF3) and ELAV like RNA binding protein 1 (ELAVL1/HuR).

YTHDC1 may affect the splicing of mRNA and its output from the nucleus [15]. YTHDF2, YTHDF3 and YTHDC2, such as YTHDF2, which promote the degradation of mRNA, can selectively bind to methylated mRNA, participate in the storage and degradation of mRNA, affect the decay process of mRNA, and may have a positive effect on human lifespan [23]. It is IGF2BP1/2/3 that maintains the stability of mRNA [24], and it is YTHDF1, YTHDF3, YTHDC2 and IGF2BP1/2/3, that promote the translation of target mRNA. Among them, YTHDF3 can assist YTHDF1 to jointly promote the translation of related mRNA [25].

## The role and significance of m^6^A methylation in ESCC

Studies have shown that m^6^A modified mRNA is maladjusted in many cancers, and its role in cancer has been gradually confirmed in vivo and in vitro, not only non-coding "writing genes", "erasing genes" and "reading genes", but also other protein factors, including oncogenes, transcription factors and signal transduction factors. the overexpression or consumption of these m^6^A-related factors may change the m^6^A modification in the tumor and interfere with the progression of cancer. The role of m^6^A regulatory factor in ESCC is shown in Table 1. Therefore, to clarify the molecular mechanism of these changes of m^6^A modified RNA and identifying the abnormal expression of m^6^A regulatory factors in clinical biopsy specimens. It is of great significance for early diagnosis of tumors, prediction of tumor prognosis and provision of new approaches of tumor treatment.

**Table 1:** The role of m^6^A regulator in ESCC

m^6^A modified type	m^6^A modification related protein	Expression	Occurrence and development of tumor	Study model	Biological Function	Ref.
m^6^A writers	METTL3	Upregulated	Promote	In vivo: human	The malignant phenotype of ESCC cells was significantly inhibited by down-regulating PI3K/AKT signal pathway	[30, 31]
m^6^A erasers	ALKBH5	Downregulated	Inhibition	–	There is a positive feedback regulation node between miR-193a-3p and ALKBH5 in esophageal cancer cells	[26]
	FTO	Upregulated	Promote	In vitro: KYSE150, Eca-109, TE-1	Up-regulation of MMP13 promotes cell proliferation and migration in esophageal squamous cell carcinoma	[32]
m^6^A readers	YTHDC2	Downregulated	Promote	In vitro: HET-1A,TE-9, Eca-109,KYSE150, EC9706	rs2416282 participates in the risk of esophageal cancer by regulating the expression of YTHDC2	[31]
	HNRNPA2B1	Upregulated	Promote	In vitro: HEEpiC, ECA109, TE10	Promoting the progress of ESCC by up-regulating the expression of fatty acid synthase ACLY and ACC1	[33]
	HNRNPC	Upregulated	Promote	-	HNRNPC may be the promoter of ESCA carcinogenesis	[34]

### m^6^A modification related protein was downregulated in ESCC.

#### ALKBH5

Previous studies [26] have found that, the expression of ALKBH5 in EC tissue decreased. Functional analysis showed that ALKBH5 could inhibit the proliferation, migration and invasion of EC cells. However, a recent study found that ALKBH5 promotes the proliferation and migration of ESCC [27]. These results contradict previous findings. The reason may be that this study did not compare the differential expression patterns between ESCC and normal esophageal tissue, but only detected the expression of ALKBH5 in ESCC. The results of the cancer genome atlas (TCGA) database through Gene Expression Profiling Interactive Analysis (GEPIA) online tool show that the overall survival time of patients with high expression of ALKBH5 is longer than that of patients with low expression, indicating that ALKBH5 plays a tumor inhibitory role in EC [28]. ALKBH5 regulates cell proliferation, migration, invasion, tumor progression, metastasis, tumorigenesis and chemotherapy resistance might by regulating m^6^A methylation.

#### YTHDC2

Yang et al. [29] through the study of the database, it was observed that the expression of YTHDC2 was downregulated in esophageal cancer. In the proliferation experiment, it was found that the low expression of YTHDC2 significantly promoted the growth of cells, suggesting that YTHDC2 may play a role as a tumor suppressor in ESCC. In the further enrichment analysis of Kyoto Encyclopedia of Genes and Genomes (KEGG) pathway, it was observed that several pathological pathways related to ESCC, including p53 signal pathway, NF-kappaB signal pathway and JAK-STAT signal pathway, were significantly rich in downregulated genes, thus promoting the proliferation of ESCC cells.

### m^6^A modification related protein expression upregulated in ESCC

#### METTL3

METTL3 is the core catalytic component of methyltransferase complex. Xia et al. [30] detected the expression of METTL3 in 207 patients with ESCC. The results of open data set and immunohistochemistry showed that the expression of METTL3 in tumor tissues was up-regulated compared with normal tissues adjacent to cancer, and the higher the expression level of METTL3 was, the worse the survival time was. In addition, it was also found that the expression level of METTL3 was an independent predictor of disease-free survival and overall survival in patients with ESCC. Another study [31] showed that small interfer RNA (siRNA) gene knockout of METTL3 inhibited the survival, colony formation, migration and invasion of EC cells, induced apoptosis, and significantly inhibited the malignant phenotype of ESCC cells by down-regulating PI3K/AKT signal pathway. In conclusion, METTL3 is a good predictor of ESCC and can be used as a potential biomarker for the prognosis of ESCC.

#### FTO

A study [32] through immunohistochemistry and data mining of 80 pairs of ESCC tissues, it was found that the expression of FTO in ESCC tissues was higher than that in adjacent normal tissues. The survival curve shows that the high expression of FTO has the trend of poor prognosis. A large number of evidence in the study confirmed that the downregulated expression of FTO can significantly inhibit the proliferation and migration of ESCC. In addition, FTO promotes cell proliferation and migration in ESCC by upregulating matrix metallopeptidase 13 (MMP13). Therefore, FTO acts as a tumor promoter in the progression of ESCC.

#### HNRNPA2B1

Guo et al. [33] found that the expression of m^6^A and its regulatory factor HNRNPA2B1 was significantly increased in ESCC tissues, and the high expression of HNRNPA2B1 was positively correlated with tumor diameter and lymph node metastasis of ESCC. In addition, functional studies have shown that HNRNPA2B1 gene knockout inhibits the proliferation, migration and invasion of ESCC. In terms of mechanism, HNRNPA2B1 promotes the progress of ESCC by up-regulating the expression of fatty acid synthase ATP citrate lyase (ACLY) and aminocyclopropane- 1-carboxylate (ACC1), indicating that HNRNPA2B1, as a carcinogenic factor, promotes the progression of ESCC by accelerating fatty acid synthesis, and may become a prognostic biomarker and therapeutic target of ESCC.

In addition, ALKBH5 and HNRNPA2B1 are effective indicators for predicting Overall Survival (OS) in patients with ESCC. High expression of HNRNPA2B1 and low expression of ALKBH5 are risk factors for ESCC survival. The combination of these two factors shows better predictive ability than using these two factors alone.

#### HNRNPC

Studies have shown that HNRNPC is overexpressed in ESCC tissues, and its expression is negatively correlated with the overall survival of patients with ESCC [34]. The double gene prognostic markers composed of ALKBH5 and HNRNPC have been proved to be a good predictor of survival outcome in ESCC.

### m^6^A methylation as a therapeutic strategy for ESCC

More and more studies have found that microRNAs [35, 36] and circular RNAs [37] can be used as potential biomarkers for prognosis, diagnosis and treatment of EC. However, due to the high morbidity and mortality of EC, it is necessary to find new anticancer drugs, such as curcumin [38], which can be potentially used in chemotherapy and chemoprevention of EC by regulating miRNAs. Allicin [39] can achieve its anticancer effect by inhibiting cell growth and inducing apoptosis.

As described in Table 1, m^6^A plays different regulatory roles in ESCC through different biological functions. In addition, a study [40] constructed and verified prognostic markers of ESCC based on m^6^A RNA methylation regulators, which may be a promising tool for predicting patient survival and provide important information for ESCC to make diagnosis and treatment strategies.

In recent years, many studies have revealed that m^6^A plays an important role in the formation of many kinds of tumors, such as breast cancer [41, 42], ovarian cancer [43, 44], cervical cancer [45], acute myeloid leukemia [46–48], glioblastomas [49–51], non-small cell lung cancer [52–55], hepatocellular cancer [56–59], gastric cancer [60], colorectal cancer [61], pancreatic cancer [62], etc. Furthermore, it is found that m^6^A modification plays a key role in tumor radiotherapy, chemotherapy and drug therapy. Table 2 summarizes the mechanism and drug resistance of m^6^A regulatory factors in related tumors, which may provide clinical reference value and significance for the treatment of esophageal cancer in the future.

**Table 2:** Mechanism and drug resistance of m^6^A regulatory factor in related tumors

Related tumors	m^6^A regulator	Roles	Study model	Mechanism	Resistance	Ref.
Breast cancer	METTL3	Oncogene	In vitro: MCF-7	METTL3, hepatitis B virus X protein binding protein (HBXIP) and miRNA let-7 g form a positive feedback loop	Tamoxifen	[41]
	ALKBH5	Oncogene	In vivo: mice	Demethylation of NANOG and increase of mRNA level		[42]
Ovarian cancer	YTHDF1	Oncogene	In vitro: SKOV3, A2780	TRIM29 may be used as an oncogene	Cisplatin	[43]
	FTO/ALKBH5	Oncogene	In vitro: PEO1	Up-regulation of Wnt/ β-catenin pathway by stabilizing FZD1	Olaparib	[44]
Cervical cancer	FTO	Oncogene	In vitro: SiHa	Regulation of β-catenin/ERCC1 axis	–	[45]
Acute myeloid leukemia (AML)	METTL3	Oncogene	In vitro: MOLM13, THP-1, MV4-11, NOMO-1, HL-60, EOL-1, KG-1, RN2c, HEL, JURKA T, LOUCY, K562	Regulating the expression of c-Myc, Bcl-2 and PTEN	–	[46]
	METTL14	Oncogene	In vivo: human	Enhanced self-renewal of hematopoietic stem cells and inhibition of bone marrow cell differentiation through SPI1-METTL14-MYB/MYC axis	–	[47]
	WTAP	Oncogene	In vitro: K562,HL-60,OCI-AML3,Ba/F3	Regulating WT1 pathway to promote cell proliferation	–	[48]
Glioblastomas (GBMs)	METTL3	Oncogene	In vivo: human	Inhibition of tumorigenesis and self-renewal / proliferation of MSCs	Y- Irradiation	[49]
	METTL14	Suppressor	In vivo: human	It is possible to target ADAM19 to inhibit tumorigenesis and self-renewal / proliferation of glioma stem-like cells (GSCs)	–	[50]
	FTO	Oncogene	In vivo: human	The inhibitory effect of drugs on FTO can inhibit the formation of m^6^A demethylation gene in glioblastoma	–	[50]
	ALKBH5	Oncogene	In vivo: mice	Demethylated FOXM1 promotes tumorigenicity of GSC	–	[51]
Non-small cell lung cancer (NSCLC)	METTL3	Oncogene	In vitro: A549, H1299, Calu6,H520,95-D, PC9,HCC827	SUMO promotes tumor growth of lysine residues K177, K211, K212 and K215 in NSCLC	Cisplatin/ Gefitinib	[52, 53]
	WTAP	Oncogene	In vitro: H1299, A549, EBC-1, HCC827,CALU-3, H661,H596, H358, H460,H1650, H1975, H1395,H292	Down-regulation of c-MET expression	Crizotinib	[54]
	YTHDF1	Suppressor	In vitro: HEK-293T, H1975, A549, NCI-H838, H1299, NCI-H1650,GLC-82, SPC-A1	regulating the translational efficiency of CDK2, CDK4, and cyclin D1	Cisplatin	[55]
Hepatocellular cance	METTL3	Oncogene	In vitro: HepG2,Huh-7,MHCC97L, HepG-2,Hepa1-6, HEK-293T,WRL68, HUVEC,SMMC-7721, Bel7402,HepG-2, WRL68, HEK-293T	Reduce the stability of SOCS2 mRNA	Sorafenib	[56, 57]
	METTL14	Oncogene	In vivo: mice	Progress in regulating miR-126 through DGCR8	Sorafenib	[58]
	YTHDF2	Oncogene	In vitro: HepG2,293T	MiR-145 regulates m^6^A level by targeting YTHDF2 mRNA 3-UTR in hepatocellular carcinoma cells	–	[59]
Gastric cancer	METTL3	Suppressor	In vitro: AGS,HGC-27, MKN-45	mediated this process occurred on the A879 locus of pri-miR-17-92	Everolimus	[60]
Colorectal cancer	YTHDF1	Oncogene	In vitro: SW480,CaCO2, HT29, RKO,DLD-1, KM12SM, HCT-116,LoVo	C-Myc promotes the expression of YTHDF1 and affects the proliferation and chemosensitivity of colorectal cancer	Oxaliplatin/ 5-Fu	[61]
Pancreatic cancer	METTL3	Oncogene	In vitro: MIA PaCa-2	METTL3 is associated with mitogen-activated protein kinase cascades, ubiquitin-dependent process and RNA splicing and regulation of cellular process	Cisplatin/ Fu / Y-Irradiation	[62]

Although many scholars have reported the study of m^6^A modification in tumor therapy, because the mechanism of tumor formation is very complex, accurate m^6^A targeting therapy needs to be explored. By changing the m^6^A level of some specific genes corresponding to mRNA in cells, it affects the expression of a series of downstream oncogenes or transcription factors. It is possible that regulating the level of m^6^A in tumor cells will become the entry point of tumor radiotherapy, chemotherapy and drug therapy [63, 64]. There is still a long way to go in the treatment of tumor in the future.

## Conclusion

In summary, with the development of biological techniques such as high-throughput sequencing, the role of m^6^A methylation in ESCC has been gradually revealed, at present, it has been found that there are abnormal expressions of METTL3, ALKBH5, FTO, YTHDC2, HNRNPA2B1 and HNRNPC in ESCC, mainly by affecting the stability of mRNA, regulating cancer cell proliferation and affecting tumor cell metastasis and invasion.

The discovery of m^6^A opens a new way for the study of epigenetics and tumor-related diseases, but the study of m^6^A modification is still in its infancy and there are still many challenges. the aim is to further study the role of epigenetic network in the occurrence and development of ESCC and to strengthen the evaluation of the safety and effectiveness of m^6^A-related regulatory factors and pathways as new targets for tumor therapy. To further explore the correlation between m^6^A and drug sensitivity and long-term prognosis of patients with ESCC, and to realize the application of m^6^A from basic research to clinical drug development as soon as possible.

## Data Availability

The datasets used and/or analyzed during the current study are available from the corresponding author on reasonable request.

## Abbreviations

m^6^A: N6-methyladenosine
mRNAs: Messenger RNAs
EC: Esophageal cancer
ESCC: Esophageal squamous cell carcinoma
METTL3: Methyltransferase like 3
WTAP: Wilms' tumor 1 associated protein
VIRMA: Virilizer like m^6^A methyltransferase
RBM15: RNA binding motif protein 15
ZC3H13: Zinc finger CCCH domain-containing protein 13
CBLL1: Casitas B-lineage lymphoma-transforming sequence-like protein 1
ALKBH5: AlkB homolog 5
FTO: Fat mass and obesity associated
YTHDF: YT521-B homology domain family
YTHDC: YT521-B homology domain containing
HNRNP: Heterogeneous nuclear ribonucleoprotein binding protein
IGF2BP2: Insulin like growth factor 2 mRNA binding protein 2
eIF3: Eukaryotic translation initiation factor
ELAVL1: ELAV like RNA binding protein 1
TCGA: The cancer genome atlas
GEPIA: Gene Expression Profiling Interactive Analysis
KEGG: Kyoto Encyclopedia of Genes and Genomes
SiRNA: Small interfer RNA
MMP13: Upregulating matrix metallopeptidase 13
ACLY: ATP citrate lyase
ACC1: Aminocyclopropane- 1-carboxylate
OS: Overall survival
